# Seasonality, morningness-eveningness, and sleep in common non - communicable medical conditions and chronic diseases in a population

**DOI:** 10.5935/1984-0063.20180017

**Published:** 2018

**Authors:** Syaron Basnet, Ilona Merikanto, Tuuli Lahti, Satu Männistö, Tiina Laatikainen, Erkki Vartiainen, Timo Partonen

**Affiliations:** 1 National Institute for Health and Welfare (THL), Department of Public Health Solutions - Helsinki - Finlândia.; 2 University of Helsinki, Department of Public Health, - Helsinki - Finland.; 3 University of Helsinki, Department of Psychology, - Helsinki - Finland.; 4 University of Eastern Finland,, Institute of Public Health and Clinical Nutrition, - Kuopio - Finland.; 5 Hospital District of North Karelia, - Joensuu - Finland.

**Keywords:** Affective Symptoms, Chronobiology Disorders, Population, Sleep Wake Disorders

## Abstract

The seasonal pattern for mood and behaviour, the behavioural trait of morningness-eveningness, and sleep are interconnected features, that may serve as etiological factors in the development or exacerbation of medical conditions. **Methods:** The study was based on a random sample of inhabitants aged 25 to 74 years living in Finland. As part of the national FINRISK 2012 study participants were invited (n=9905) and asked whether the doctor had diagnosed or treated them during the past 12 months for chronic diseases. **Results:** A total of 6424 participants filled in the first set of questionnaires and 5826 attended the physical health status examination, after which the second set of questionnaires were filled. Regression models were built in which each condition was explained by the seasonal, diurnal and sleep features, after controlling for a range of background factors. Of the chronic diseases, depressive disorder was associated with longer total sleep duration (*p*<.0001) and poor sleep quality (*p*<.0001). Of the measurements for health status assessment, none associated with sleep features, but systolic blood pressure yielded significant (*p*<.0001) associations with both seasonal and diurnal features at large. **Conclusion:** Sleep quality was the most sensitive probe in yielding associations with chronic diseases in this population-based study. The seasonal variations in mood and social activity, and the ease in getting up and tiredness in the morning were the most sensitive probes in yielding associations with blood pressure and waist circumference. Assessment of sleep quality, seasonal and diurnal features provides thus added value for health surveys of the general population.

## INTRODUCTION

Circadian-clocks create an intrinsic time-tracking system that measures the passage of time in our tissues, generates and maintains the endogenous circadian rhythms^1^. These clocks, enable organisms to adapt their behavior (such as: feeding, sleeping, and mating) and physiological functions (such as: cardiovascular activity, endocrine functions, body temperature and hepatic metabolism) with the environmental changes caused by the rotation of the earth along with its trajectory around the sun^2^^-^^4^. Disruptions in these circadian alignments with environmental entrainment factors may manifest sleep disorders, hormonal imbalances, chronic diseases, and reduces life-span^5^.

Measures of these circadian rhythms, could be an important determinant of the health status. For example, sleep problems are linked with health complaints in shift workers, and among those with existing chronic illnesses^6^^-^^10^. Further, to certain extent of a problem, reoccurring seasonal variations in mood and behavior (seasonality) seems to impair well-being by causing atypical depressive symptoms, such as carbohydrate craving, overeating, weight gain, hypersomnia, lack of energy, and decreased libido that are common atypical symptoms in seasonal affective disorder^11^^,^^12^. In a similar way, the extreme traits of morningness-eveningness (chronotype), which is based on the intrinsic tendency of individuals to wake-up and fall-asleep at particular times of the day: possibly affects sleep schedules, contributes relapses of an illness, disturbs physical activity patterns, delays cognitive performance, affects endocrine functions, and overall behavioral choices^13^^-^^16^.

Circadian alignment with environmental entrainment factors contribute to biological changes that may catalyse to be an etiological factor in the development and exacerbation of disease and conditions including cardiovascular and metabolic disorder^17^. Such factors include, sleep, seasonality and chronotype that are interconnected features, but if disrupted, may compromise the health status^18^. To elaborate, poor sleep is suggested to elevate inflammatory markers like cytokines, and increase oxidative stress^10^^,^^19^. A shortage of sunlight during winter months may lead to inadequate resetting of the circadian clocks, and thereby links with the etiology of chronic diseases and pronounced seasonality^20^.

Moreover, the duration and intensity variation in light exposures influences the relapse of an illness throughout the year based on individual chronotype^18^. It is also suggested that eveningness associates with or even predisposes a range of common chronic diseases which are not limited to bronchial asthma, cardiovascular diseases, depressive disorders, seasonal affective disorder, sleep disorders, spinal diseases, and type-2 diabetes, but also a range of common medical conditions such as: anxiety, burnout, obesity, and substance use^21^^-^^34^.

Moreover, non-communicable chronic diseases impose the largest public health burden globally. This burden accounted for 68% (38 million) of the world’s 56 million deaths in the year of 2012^35^, however, it extends beyond mortality through its impact on health with larger financial consequences. One possible etiological mechanism for these diseases is a possible misalignment between a time-givers (Zeitgebers) and the circadian rhythms like sleep-wake cycle. Hence, by understanding the relationship between chronic diseases and circadian systems biology for sleep characteristics, chronotype and seasonality, a new perspective on the course, treatment, and outcome of these diseases could be achieved.

Thus, in the present study, we have analyzed the association of three indicators of circadian alignment with individual (sleep characteristics and chronotype) and environmental factors (seasonality) with common chronic non-communicable diseases and medical conditions at population level. In addition, we studied how routine objective health examination measurements were related to these three indicators.

## METHODS

### Participants

The national FINRISK-study, is a Finnish population-based health-examination survey conducted in every five-years since 1972 in Finland. For the present study, in 2012, a random sample of 10,000 adults aged 25 to 74-years living in five geographical areas of Finland, stratified by sex and age, were derived from the population information system of the Finnish national population register center. At start of the study, there were 9905 individuals alive and living in Finland, and they were invited to participate.

A total of 6424 participants filled in the first-set of questionnaires, and they reported their total-sleep duration and sleep quality. A total of 5826 participants attended the health examination, after which they were asked to fill in and send back the second set of questionnaires where they reported their seasonal variations in mood and behavior, and their diurnal preference in behavior. The participant rate was 64%.

### Assessment

The study included two-sets of self-administered questionnaires that included structured questions on socioeconomic factors, medical history, health behavior, psychosocial factors, and a physical examination of health status accompanied with laboratory test measurements. These were sent by regular-mail together with an invitation to attend a health status examination in a local health care center or other facility near to the participant’s residence. The participants filled in the questionnaire at home and returned it at the health status examination where it was checked by the staff and, if needed, completed with the participant. At the health status examination, the participants were given another questionnaire which they returned via regular mail to the institute.

Health examination measurements in the current study included height (cm), weight (kg), body-mass index (BMI, kg per m^2^), systolic and diastolic blood pressures (mean of three measurements in mmHg), waist circumference (cm), and the daily energy consumption (basic metabolic rate as assessed with bioimpedance measurement [TBF300 MA, Tanita, Arlington Heights, IL, USA] in kcal per day). These physical measurements were made at the health status examination in a local health care center or other facility near the participant’s residence. They were made by nurses who were trained by the staff at the institute for two weeks before the start of the study.

Seasonal variations were assessed with a modified self-rating Global Seasonality Score (GSS), the key subscale of the Seasonal Pattern Assessment Questionnaire (SPAQ)^36^. It consists of the answers given to the six items asking, to what degree the participant’s sleep duration, social activity, mood, weight, appetite, and energy level change with the seasons? Each item was scored on a Likert-like scale as 0 (no variation) to 3 (marked variation). The behavioral trait of morningness-eveningness was assessed with the six-items (items 4, 7, 9, 15, 17 and 19) derived from the original nineteen-items Morningness-Eveningness questionnaire (MEQ)^37^.

These six items explained 83% of the variation in the original MEQ sum score, with their Cronbach’s alpha of .80^38^.The item-4 asks the ease in getting up in the morning, and it was categorized into: not easy to get up (not at all or not very easy) *vs.* easy to get up (quite easy or very easy). The item-7 asks the feeling of tiredness in the morning, and it was categorized into: feeling tired during the first half-hour after having woken in the morning (very tired or quite tired) *vs.* feeling rested (quite rested or very rested). The item-9 asks the early morning performance in some physical exercise, and it was categorized into: feeling difficult performing in morning hours (would feel very difficult or would feel quite difficult) vs. feeling in good condition (would be in moderate condition or would be in good condition).

The item-15 asks the alternative time slots for hard physical work if free to plan the day, and it was categorized into: morning-hour choices (11 am to 1 pm or 8 to 10 am) vs. evening-hour choices (7 to 9 pm or 3 to 5 pm). The item-17 asks the choice for working hours as a 5-hour block, and it was scored on a Likert-like scale and coded as: 1 (five consecutive hours starting between 5 pm and 4 am), 2 (five-consecutive hours starting between 2 and 5 pm), 3 (five-consecutive hours starting between 9 am and 2 pm), 4 (five-consecutive hours starting between 8 and 9 am), or 5 (five-consecutive hours starting between 4 and 8 am). The item-19 asks the opinion about being a morning or evening type of person, and it was categorized into: evening types (definitely evening type or more evening than morning type) *vs.* morning types (more morning than evening type or definitely morning type).

Sleep variables were subjectively reported for total sleep duration and sleep quality. Total sleep duration was based on the response about the average sleep duration (in hours and minutes) in 24 hours. There was a single item asking about sleep quality: “Do you think that you sleep enough?”: “Yes, almost always; Yes, often; Seldom or nearly never; I cannot say”. Sleep duration was asked with three separate items: “How many hours do you sleep on average a) at night, b) per day (night sleep plus daytime naps as together)?”, “What is your usual bedtime (when you are going to go to bed and have sleep) a) on workdays/weekdays, b) on free days/in weekends?”, and “What is your usual wake-up time (when you are not going to go back to bed) a) on workdays/weekdays, b) on free days/in weekends?”.

### Covariates

Background information covariates were age (in years), gender (male or female), civil status as living with somebody (either married, cohabitating or registered partnership) vs. alone (either single, separated or divorced, or widow), education as low (less than 4 years of high school), medium (either high school only or 1 to 3 years post high school) or high (4 or more years post high school) levels, region as living in North Karelia and Kuopio, North Savo, Turku and Loimaa, Helsinki and Vantaa, or in Oulu.

Lifestyle covariates were smoking as smokers (either smoked daily or occasionally) *vs.* non- smokers (smoked not at all), alcohol consumption as alcohol consumption (at least once or more than once a month) *vs.* no alcohol consumption (no alcohol consumption at all), and physical activity as regular exercise (either several times a week or at least 3-4 hours per week or) *vs.* no exercise.

The participants were asked if (yes or no) the doctor had diagnosed or treated them in the past 12 months for the following common non-communicable medical conditions and chronic diseases: hypertension, high cholesterol, cardiac insufficiency, angina pectoris, diabetes, cancer, bronchial asthma, chronic obstructive pulmonary disease (COPD), gallstones, rheumatoid arthritis, other joint diseases, degenerative arthritis of the back, depressive disorder, other mental disorders, renal failure, and proteinuria. These sixteen variables were the primary outcome measures for the study.

### Statistical analysis

Group differences were calculated, and the statistical significance was tested. Univariate and binary logistic regression models with the health examination measurements and the medical conditions and chronic diseases as the dependent variable, and the GSS items, sleep variables and MEQ items as the independent explanatory variables were generated, after controlling for BMI and the background information and lifestyle covariates (age, gender, civil status, education level, region, smoking, alcohol consumption, and physical activity).

No seasonal variation in the respective GSS item, good sleep quality, easy getting up in the morning, well rested, good condition, morning hours, and morning type categories were used as the reference. The level of significance was adjusted for the number of statistical tests we calculated (294 tests) by using the conservative Bonferroni correction, and thus the *p*-values of less than .00017 were considered as significant. The data were analyzed with the IBM SPSS statistics 21 software.

### Ethics

The data collection was conducted according to the guidelines of the Declaration of Helsinki and international ethical standards.

The Ethics Committee of the Hospital District of Helsinki and Uusimaa (HUS) approved the research protocols. All the participants gave a written informed consent.

## RESULTS

The 6424 participants (3383 women, 3041 men) were aged 51 years on average. Of the participants, 19% slept for less than 7 hours, 63% slept for 7 to 8 hours, and 17% slept for more than 8 hours per night. Altogether, 14% of the participants (8% of women, 6% of men) reported poor sleep quality. About 70% of the participants reported seasonal variations in sleep duration (73%), social activity (71%), mood (72%) or energy level (75%), and about 40% those in weight (46%) or appetite (43%).

Of the 4689 participants (2562 women, 2127 men) with the complete assessment of seasonality, 168 (3.58%) had the variations to the extent equal to seasonal affective disorder, 429 (9.15%) equal to subsyndromal seasonal affective disorder, and 4092 (87.27%) had normal seasonality. Of the 4414 participants (2450 women, 1964 men) with the complete assessment of chronotype, 595 (3.5%) were evening types (“night owls”), 1884 (42.7%) were intermediate types, and 1935 (43.8%) were morning types (“morning larks”). Of the health examination measurements, systolic blood pressure was significantly (*p*<.0001) associated with all the six items of seasonality, except that of weight, and with all the six items of morningness-eveningness, except that of choice for working hours (see Table 1 & Table 2).

**Table 1:** Correlation coefficient (r) values for the seasonality, sleep, and morningness eveningness in relation to the five health measurements.

Measurement	Seasonality (GSS items)							Sleep
	Sleep length	Social activity	Mood	Weight	Appetite	Energy level	Sleep duration	Sleep quality
Body-mass index	-.008	-.001	.000	-.003	-.004	-.004	-.053**	.012
Systolic blood pressure	.056****	.109****	.112****	-.013	.068****	.094****	-.004	-.008
Diastolic blood pressure	.032*	.055****	.062****	-.031*	.027	.040**	-.029	-.020
Waist circumference	.065****	.077****	.079****	-.148****	-.035*	.052***	-.025	-.010
Energy consumption per day	.006	.007	.019	-.224****	-.114****	-.011	-.016	.003

Abbreviations: GSS=Global Seasonality Score Questionnaire. Covariates of the partial correlations include age and gender. Reference group: GSS items=no seasonal variation in the respective item; Sleep quality=good sleep. Significance indicated as *p*=

* <0.05,

** <0.01,

*** <0.001,

**** <0.0001.

**Table 2:** Correlation coefficient (r) values for morningness-eveningness in relation to the five health measurements.

Measurements	Morningness-eveningness (MEQ items)					
	Not easy to get up	Feeling morning tiredness	Poor early morning performance	Evening hours choice	Evening choice of consecutive hours	Evening type
Body-mass index	-.026	-.037*	-.006	-.005	.013	-.010
Systolic blood pressure	.146****	.204****	.128****	.076****	.010	.104****
Diastolic blood pressure	.104****	.128****	.131****	.038*	-.010	.085****
Waist circumference	.086****	.116****	.059***	.061****	.021	.025
Energy consumption per day	.045**	.058***	.009	.055***	.003	.032

MEQ=Morningness-Eveningness Questionnaire Significance indicated as *p*=

* <0.05,

** <0.01,

*** <0.001,

**** <0.0001

In addition, diastolic blood pressure yielded significant associations more with diurnal features, whereas the waist circumference more with seasonal ones, and the daily energy consumption per day was associated with the seasonal variations in weight and appetite.

Concerning the medical conditions and chronic diseases, depressive disorder was significantly associated with poor sleep quality (ß=.459, *p*<.001) and longer total sleep durations (ß=3.33, *p*<.001) (see Table 3). Depressive disorder associated with the seasonal variations in mood (OR=.46, *p*<.05) and appetite (OR=.51, *p*<.01). Poor sleep quality was reported in almost all the chronic diseases with the highest significant odds for gallstones (OR=21.59, *p*<.01) and COPD (OR=4.89, *p*<.01).

**Table 3:** Odds ratios with 95% confidence intervals, or beta values with standard errors, for the seasonality, sleep and morningness-eveningness in relation to the 16 outcome measures.

Condition	Seasonality (GSS items)							Sleep
	Sleep length	Social activity	Mood	Weight	Appetite	Energy	Sleep quality	Sleep duration
	OR (95% CI)							Beta (s.e.)
Hypertension	.83 (.59-1.17)	1.19 (.82-1.71)	1.14 (.77-1.66)	.99 (.71-1.39)	.86 (.61-1.21)	.80 (.53-1.19)	1.01 (.62-1.65)	-.006(.075)
High cholesterol	.923 (.66-1.28)	.95 (.66-1.36)	1.05 (.72-1.53)	1.14 (.82-1.58)	1.02 (.73-1.43)	1.01 (.69-1.49)	1.22 (.77-1.93)	-.07(.073)
Cardiac insufficiency	.615 (.26-1.44)	1.18 (.50-2.79)	.99 (.38-2.53)	.78 (.36-1.68)	1.20 (.53-2.67)	.96 (.36-2.52)	3.55 (1.37-9.14) **	-.017(.173)
Angina pectoris	.82 (.34-1.97)	.41 (.14-1.15)	1.12 (.43-2.88)	1.18 (.53-2.64)	.77 (.33-1.76)	.66 (.22-1.93)	1.60 (.52-4.89)	-.085(.400)
Diabetes	1.23 (.73-2.06)	1.20 (.68-2.08)	1.01 (.55-1.83)	1.17 (.69-1.97)	.72 (.42-1.23)	.78 (.41-1.45)	1.11 (.47-2.56)	.131(.113)
Cancer	.97 (.39-2.35)	1.48 (.56-3.90)	.82 (.28-2.36)	2.47 (.92-6.58)	2.40 (.82-6.97)	.26 (.07-.84) *	2.00 (.54-7.38)	.168(.207)
Bronchial asthma	.81 (.46-1.40)	.77 (.41-1.43)	.81 (.43-1.52)	.86 (.50-1.44)	1.18 (.68-2.01)	1.51 (.80-2.83)	2.41 (1.30-4.47) **	.227(.113) *
COPD	.71 (.24-2.10)	1.81 (.59-5.48)	.84 (.25-2.75)	.61 (.22-1.64)	.99 (.35-2.75)	.43 (.11-1.63)	4.90 (1.26-18.93) *	.565(.207) **
Gallstones	1.81 (.22-14.86)	.48 (.03-6.00)	.45 (.02-7.77)	3.51 (.53-22.9)	1.57 (.23-10.64)	.43 (.023-8.01)	21.60 (2.76-168.8) **	-.014(.415)
Rheumatoid arthritis	.83 (.33-2.06)	1.30 (.49-3.40)	1.65 (.60-4.51)	.66 (.25-1.70)	3.18 (1.06-9.55) *	.82 (.29-2.28)	.79 (.18-3.38)	-.176(.199)
Other joint disease	.70 (.45-1.08)	1.26 (.79-1.98)	.82 (.50-1.32)	.66 (.44-.98) *	.86 (.57-1.28)	1.05 (.63-1.71)	1.16 (.65-2.06)	.066(.090)
Degenerative arthritis	.88 (.62-1.24)	1.07 (.73-1.57)	.58 (.38-.87) **	.82 (.58-1.13)	1.20 (.85-1.68)	.86 (.56-1.30)	1.69 (1.09-2.60) **	.081(.075)
Depressive disorder	1.09 (.62-1.90)	1.00 (.51-1.92)	.46 (.20-.99) *	1.09 (.65-1.83)	.51 (.29-.87) **	.68 (.30-1.50)	3.33 (1.82-6.08) ****	.459(.115) ****
Other mental disorder	1.80 (.76-4.22)	1.59 (.57-4.37)	.80 (.25-2.51)	1.09 (.44-2.63)	.60 (.24-1.49)	.45 (.12-1.65)	1.71 (.56-5.22)	.562(.184) **
Renal failure	.26 (.01-7.42)	1.43 (.12-15.90)	.80 (.04-13.80)	4.00 (.29-54.82)	3.89 (.19-77.59)	.55 (.02-12.92)	14.43 (.53-387.49)	.67(1.670)
Proteinuria	.25 (.03-2.16)	2.16 (.57-8.10)	.72 (.12-4.10)	1.11 (.32-3.79)	.48 (.13-1.69)	.34 (.03-3.46)	2.43 (.57-10.21)	.981 (-.007)

Abbreviations: GSS=Global Seasonality Score; MEQ=Morningness-Eveningness Questionnaire; OR=odds ratio; CI=confidence interval; s.e.= standard error; COPD=chronic obstructive pulmonary disease. Reference groups: GSS items=no seasonal variation in the respective item; Sleep quality=good sleep; Covariates of the regression models include the body-mass index, age, gender, region, civil status, education level, alcohol intake, and smoking. Significance indicated as *p*=

* <0.05,

** <0.01,

***<0.001,

**** <0.0001.

Morningness-eveningness in relation to the outcome is reported in Table 4. Bronchial asthma was significantly associated with morning tiredness (OR=1.69, *p*<.05) and being an evening type (OR=.46, *p*<.01 (see Table 4 for details).

**Table 4:** Odds ratios with 95% confidence intervals, or beta values with standard errors, for morningness-eveningness in relation to the 16 outcome measures.

Condition	Morningness-eveningness (MEQ items)					
	Not easy to get up	Having morning tiredness	Poor early morning performance	Evening hours choice	Evening choice of consecutive hours	Evening type
	OR (95%CI)	OR (95%CI)	OR (95%CI)	OR (95%CI)	Beta (S.E)	OR (95%CI)
Hypertension	.93 (.59-1.46)	1.39 (.95-2.00)	1.01 (.73-1.37)	1.16 (.76-1.77)	.036(.197)	.80 (.57-1.10)
High cholesterol	1.29 (.82-2.03)	.84 (.57-1.22)	.89 (.65-1.20)	1.50 (.99-2.26) *	.027(.199)	.78 (.57-1.07)
Cardiac Insufficiency	1.15 (.38-3.44)	1.65 (.68-3.97)	.31 (.13-.70) **	1.65 (.62-4.38)	.056(.497)	1.58 (.72-3.45)
Angina Pectoris	1.32 (.36-4.75)	.43 (.14-1.30)	.92 (.41-2.01)	1.84 (.67-5.06)	-.110(.482)	.82 (.36-1.82)
Diabetes	1.17 (.58-2.37)	1.24 (.69-2.20)	.92 (.56-1.50)	1.65 (.88-3.07)	.399(.330)	.70 (.42-1.16)
Cancer	1.00 (.28-3.51)	1.37 (.48-3.85)	.57 (.24-1.33)	.41 (.08-1.89)	.768(.684)	1.54 (.66-3.56)
Bronchial asthma	.63 (.30-1.27)	1.69 (.98-2.92) *	1.79 (1.08-2.92) *	.82 (.41-1.61)	-.350(.288)	.46 (.27-.77) **
COPD	1.37 (.25-7.51)	.37 (.09-1.42)	1.60 (.61-4.17)	1.25 (.31-4.95)	-0.64(.579)	.85 (.31-2.32)
Gallstone	4.65 (.26-80.53)	.01 (0-0.47) *	3.02 (.43-21.08)	1.25 (.08-17.45)	-2.13(1.02) *	1.46 (.22-9.53)
Rheumatoid arthritis	.67 (.20-2.18)	1.66 (.62-4.40)	1.65 (.67-4.01)	2.12 (.73-6.11)	.155(.537)	1.19 (.47-3.00)
Other joint diseases	1.13 (.63-2.02)	.70 (.43-1.12)	1.13 (.77-1.65)	1.38 (.83-.29)	-.034(.245)	.96 (.64-1.41)
Degenerative arthritis	1.25 (.79-1.97)	.78 (.53-1.15)	1.34 (.97-1.83)	1.24 (.82-1.86)	.161(.195)	.99 (.71-1.37)
Depressive disorder	.90 (.43-1.86)	1.27 (.71-2.24)	1.35 (.82-2.20)	.79 (.37-1.65)	-.370(.305)	.94 (.55-1.58)
Other mental disorder	1.26 (.31-5.14)	.49 (.15-1.49)	.80 (.34-1.82)	2.93 (1.07-8.04) *	.111(.488)	1.08 (.44-2.63)
Renal failure	-	14.43 (.53-387.4)	.65 (.06-6.62)	-	1.089(1.500)	.70 (.06-7.22)
Proteinuria	.77 (.11-5.14)	.77 (.17-3.32)	.80 (.23-2.69)	1.04 (.19-5.44)	-.043(.721)	1.27 (.35-4.52)

Abbreviations: Reference group MEQ items=Morningness-Eveningness Questionnaire: easy to get up, feeling rested in the morning, feels good about early morning performance, morning hour choice, morning choice of consecutive hours, and morning type; OR=odds ratio; CI=confidence interval; s.e.=standard error; COPD=chronic obstructive pulmonary disease. Covariates of the regression models include the body-mass index, age, gender, region, civil status, education level, alcohol intake, and smoking. Significance indicated as *p*=

* <0.05,

** <0.01,

***<0.001,

****<0.0001.

## DISCUSSION

In the current study, we analyzed, whether key features of seasonality, morningness-eveningness and sleep were associated with the presence of common non-communicable medical conditions or chronic diseases and assessed their independent contributions. This study is, to our best knowledge, the first one to report such associations on population level. This study reveals two major findings as follows:

First, of the 16 medical conditions and chronic diseases assessed, depressive disorder and bronchial asthma yielded the greatest number of associations with the explanatory variables. Of these, the associations of sleep features with depressive disorder remained significant after adjustment for multiple testing. Thus, this current finding, which emerged from the single-item analysis corroborated the earlier reports on the association of depressive disorder with sleep^39^.

This finding fails to reproduce earlier reports with the association of depressive disorder with global seasonality^40^ or chronotype^32^ which both thus appear to be more complex constructs than the self-reported sleep quality or duration. In contrast, there are epidemiological and clinical studies, reporting strong correlation between sleep disorders and depression^41^^,^^42^. For example, in a large community-based population study, the self-reported short sleep duration and increased sleep disturbances were independently associated with increased cortisol secretion suggesting chronic stress^43^. In bronchial asthma, we found significant associations with eveningness, this result reciprocates with Merikanto et al.^29^. Furthermore, there are others reporting associations of poor sleep quality with breathing abnormalities in respiratory diseases^44^^-^^46^, while not many exist to validate the current finding.

Second, of the five health examination measurements we analyzed, systolic blood pressure that has its approximate 24-hour (i.e., circadian) rhythm was significantly associated with a number of the explanatory variables, except those of sleep features. Interestingly, energy consumption per day, as assessed with bioimpedance measurement, was significantly associated with the seasonal variations in weight and appetite, but not with the remaining seasonal variations. Thus, this finding fits in the view that the global seasonality is a mixture of two components, as being evidenced by loadings on two factors^47^^,^^48^, where the one includes weight and appetite and the other includes the remaining.

There are limitations that need to be addressed for the current study. First, the data on the medical conditions and chronic diseases, seasonality, morningness-eveningness, and sleep length and quality were based on self-reports, which were provided as part of the health examination study. Thus, some assessment noise and misclassification may have occurred. However, the health examination measurements were assessed with objective methods. Second, the study design was cross- sectional, leaving the causal relationships between the outcome and explanatory variables unanswered. Third, the associations should be interpreted with caution due to a small number of cases for some outcomes. Further, earlier research suggests that the direct genetic effect on the chronotype equals to 40% to 70%, while the rest is influenced by environmental factors such as age, physical activity, meal time and melatonin^49^. These factors need to be addressed in further studies.

Despite limitations, there are also strengths in the current study. First, it was based on a large population-based data, covering large areas of the country, due to which the results are generalizable, and the potential risk of recruitment bias is reduced. Second, the medical conditions and chronic diseases which were based on the subjective report of the participants were also clinically verified by the diagnoses assessed or treatment provided by a medical doctor. Third, the present study compares the circadian alignment with environmental entrainment factors together, while the other studies assessed the same circadian alignment independently with the same population study^39^^,^^40^^,^^50^.

## CONCLUSION

To conclude, we herein analyzed the seasonality, morningness-eveningness, and sleep features simultaneously in the same statistical model which we controlled for a range of confounding factors and whose results we adjusted for multiple testing. We found that poor sleep quality contributed most to the outcome in case of depressive disorder but was not associated with any of the health examination measurements.
